# *Ichnocarpus frutescens* Ameliorates Experimentally Induced Convulsion in Rats

**DOI:** 10.1155/2014/434179

**Published:** 2014-10-29

**Authors:** Narendra Kumar Singh, Damiki Laloo, Debapriya Garabadu, Tryambak Deo Singh, Virendra Pratap Singh

**Affiliations:** ^1^Department of Medicinal Chemistry, Faculty of Ayurveda, Institute of Medical Sciences, Banaras Hindu University, Varanasi 221 005, India; ^2^Department of Pharmaceutics, Indian Institute of Technology, Banaras Hindu University, Varanasi 221 005, India

## Abstract

The present study was carried out to evaluate the anticonvulsant activity and probable mechanism of action of the methanol root extract from *I. frutescens* (MEIF) using different experimental animal models. Anticonvulsant activity of the single dose of MEIF (100, 200, and 400 mg/kg, p.o.) was evaluated in maximal electroshock- (MES-), pentylenetetrazole- (PTZ-), and isoniazid- (INH-) induced convulsions models in rats. The levels of *γ*-amino butyric acid (GABA), glutamate, GABA-transaminase (GABA-T) activity and oxidative stress markers were measured in pretreated rat's brain homogenate to corroborate the mechanism of observed anticonvulsant activity. MEIF (200–400 mg/kg, p.o.) protected the animals in all the behavioral models used. Pretreatment of MEIF (200–400 mg/kg, p.o.) and diazepam (1.0 mg/kg, i.p.) to the animals in INH-induced convulsion model showed 100% and 80% protection, respectively, as well as significant restoration of GABA and glutamate level in the rat's brain. MEIF and vigabatrin (50 mg/kg, i.p.) reduced the PTZ-induced increase in the activity of GABA-T (46%) in the brain. Further, MEIF reversed the PTZ-induced increase in lipid peroxidase (LPO) and decrease in reduced glutathione (GSH), catalase (CAT), and superoxide dismutase (SOD) activities. The findings of this study validate the anticonvulsant activity of *I. frutescens*.

## 1. Introduction

Epilepsy is the result of abnormal, hypersynchronous neuronal activity (a lot of irregular neuronal activity) [1], affecting approximately 50 million of the people worldwide with an approximate estimation of at least 7 million in India [2, 3]. The term epilepsy is used for the brain disorder, characterized by occurrence of spontaneous seizures (convulsion, sensory disturbances, or loss of consciousness resulting from the abnormal electrical discharges in the brain), due to the imbalance of complex neurotransmitter systems [4]. Clinically, epilepsy can be classified into two broad categories, namely, partial seizures (discharge remains localized) and generalized seizures (involves the whole brain). Either form is classified as simple (if consciousness is not lost) or complex (if consciousness is lost). Two important subcategories of generalized seizures are tonic clonic seizures (grand mal) and absence seizures (petit mal) [5]. Pharmacotherapy of epilepsy includes phenytoin, carbamazepine, valproate, ethosuximide, phenobarbital, benzodiazepines, vigabatrin, and gabapentin; however their use is restricted based on certain severe adverse effects such as hypersensitivity, sedation, megaloblastic anaemia, and teratogenic effect [5]. Thus, it is a prerequisite to search for an alternative and effective source as an antiepileptic drug with a good safety profile and lesser side effects. Recently, herbal medicines gained critical attention as a good source of alternate medicine for the treatment of various disorders due to lesser side effects [6]. It has been reported that several medicinal plants such as* Abelmoschus angulosus* Wall. ex Wight & Arn. (Malvaceae),* Allium sativum* L. (Amaryllidaceae),* Cinchona officinalis* L. (Rubiaceae),* Plumbago zeylanica* L. (Plumbaginaceae), and* Egletes viscosa* (L.) Less. (Asteraceae) are used since time immemorial for the treatment of epilepsy in the Indian system of traditional medicine [7].

*Ichnocarpus frutescens *(L.) R. Br. (Apocynaceae) is a woody climber, distributed in India, ascending to an altitude of up to 4000 ft above sea level [8]. Traditionally, most of the ethnic communities in India used this plant for the treatment of diabetes, skin diseases, epilepsy, chronic nervous diseases, debility, impotence, abdominal distention, and gall bladder stone [9–12].* I. frutescens* root is one of the important ingredients of the Ayurvedic formulations “Ashwagandharishta” and “Swalpachaitasa Ghrita,” which are indicated for the treatment of epilepsy and other ailments [13]. The important phytoconstituents reported from this plant are *α*-L-rhamnopyranosyl-(1→4)-*β*-D-glucopyranosyl-(1→3)-*α*-amyrin [14], *α*-amyrin, lupeol, *β*-sitosterol, friedelin, epi-friedelinol [15], apigenin, luteolin [16], kaempferol, kaempferol-3-galactoside, ursolic acid acetate [17], quercetin, and quercetin-3-O-*β*-D-glucopyranoside [18]. Pharmacologically, various activities have been reported from this plant which include antiurolithiatic, hepatoprotective, anti-inflammatory, antipyretic, analgesic, antidiabetic, anticancer, antihyperlipidemic, and antioxidant activities [12]. Traditionally, the root of this plant is used in the treatment for epilepsy; however no scientific information has been reported so far to validate the anticonvulsant activity in experimental animals. Hence, the present investigation was designed to scientifically validate the antiepileptic activity of the methanol extract from the root of* I. frutescens* (MEIF) using MES-, PTZ-, and INH-induced convulsion models in rats.

Amino acid transmitters particularly play an important role in the pathogenesis of epileptic conditions [19, 20]. It is well accepted that changes in amino acids concentration in the brain following convulsions include decrease in inhibitory amino acids level such as GABA, taurine, and alanine and an increase in the concentration of the excitatory amino acids level such as aspartate and glutamate [21]. Thus, the present study was designed to explore the effect of MEIF on the level of GABA and glutamate in the INH-induced model of epilepsy. Further, the effect of MEIF was investigated on the GABA-T activity and oxidative stress in the PTZ-induced convulsion model.

## 2. Materials and Methods

### 2.1. Chemicals and Reagents

Pentylenetetrazole, isoniazid, gamma aminobutyric acid, glutamate, diazepam (D-907), phenytoin (PHR1139), and vigabatrin were purchased from Sigma Ltd., USA. ELISA kit (MBS939900; MyBioSource, Inc., San Diego, CA, USA) was used for the estimation of GABA-T activity.

### 2.2. Plant Material

Plant material was collected in the month of December (2011) from the medicinal garden of Rajiv Gandhi South Campus, Banaras Hindu University, Barkachha, Mirzapur, UP, India. The identification of the plant material was done by Dr. Subir Bandyopadyay at the Botanical Survey of India, Howrah, West Bengal, India (plant identification letter: CNH/104/2012/Tech. II/950). For future reference a voucher specimen (number PRL-02) of the plant material has been deposited in the Department of Medicinal Chemistry, Faculty of Ayurveda, Institute of Medical Sciences, Banaras Hindu University, Varanasi.

### 2.3. Preparation of Plant Extract

Roots of* I. frutescens* were separated from the aerial parts and shade-dried until a constant weight was obtained. The roots were then pulverized into coarse powder and sieved by 20# sieve. The coarse powdered drug (475 g) was extracted with methanol (2.0 l) by cold maceration process for 10 days. After 10 days the content was filtered and the filtrate obtained was concentrated under reduced pressure in rotary evaporator (Perfit India, Pvt. Ltd., India) below 60°C. Methanol extract of* I. frutescens* (MEIF) (5.26% w/w) obtained was kept in desiccator for several days to completely remove the traces of solvent.

### 2.4. Animals

Inbred Charles Foster rats (120–150 g) of either sex were obtained from the Central Animal House of Banaras Hindu University, Varanasi. The experimental study was performed after receiving necessary approval from the Institutional Animal Ethical Committee (IAEC), Institute of Medical Sciences, Banaras Hindu University, Varanasi (Dean/13-14/CAEC/193). All the animals were housed and maintained under standard laboratory conditions in polypropylene cages at constant room temperature (22.0 ± 3.0°C), relative humidity (50 ± 10%), and 12 : 12 h dark and light cycle. The animals were allowed to acclimatize to the environment of the laboratory for 7 days before the commencement of experiments. Animals were fed with commercial rat feed (Amrut, Pvt. Ltd., Pune, India) and water* ad libitum*. Each animal was used for one seizure experiment only. Fasting of the animals (with free access to water* ad libitum*) for 18 h prior to the experiment was done in all the tested anticonvulsant models.

### 2.5. Acute Toxicity Studies

The acute oral toxicity study of MEIF was performed according to the Organization for Economic Co-Operation and Development (OECD)—425 guidelines. Single dose of MEIF 2000 mg/kg, p.o., was administered in 24 h fasted rats (*n* = 5) and rats were observed at 0, 30, 60, 120, 180, and 240 min and then once a day for the next 14 days for any signs or symptoms of toxicity or abnormalities. The number of rats that survived at the end of the study period was recorded [22].

### 2.6. Evaluation of Anticonvulsant Activity of MEIF in Different Animal Models of Convulsion

#### 2.6.1. Maximal Electroshock- (MES-) Induced Convulsion

The maximal electroshock seizure (MES) was tested in animals through transauricular electrodes attached bilaterally to the animal ear. Electroconvulsive shock producing an alternating current stimulus (150 mA, 50 Hz, 0.2 s duration) was delivered through ear-clip electrodes to induce hind limb tonic extension (HLTE) in rats. The electrodes were moistened with a drop of electrolyte solution prior to delivery of the electroshock [23]. Animals were divided into five groups of six animals in each group (*n* = 6). Group I (negative control) served as untreated group and received only carboxymethyl cellulose (0.5% CMC, p.o.). Group II (standard treated group) received single dose of phenytoin (25 mg/kg, i.p.), whereas Groups III, IV, and V (MEIF treated groups) received the single dose of the MEIF 100, 200, and 400 mg/kg, p.o., respectively. All the animals received electric shock stimulation 30 min after the treatment with standard drug and graded dose of MEIF. After electrical stimulation, the occurrence and duration of HLTE and incidence of mortality were noted. The criterion for the evaluation of anticonvulsant activity is the ability to prevent HLTE or reduction in the duration of HLTE [24].

#### 2.6.2. Isoniazid- (INH-) Induced Convulsion

In INH-induced convulsion model, animals were randomly divided into six groups comprising six animals in each group (*n* = 6). Group I (normal control) received only vehicle (0.5% CMC, p.o.). Group II (negative control) was administered with INH (250 mg/kg, i.p.) only. Group III (standard treated group) received standard drug diazepam (1.0 mg/kg, i.p.). Groups IV, V, and VI (MEIF treated groups) were administered with MEIF 100, 200, and 400 mg/kg, p.o. Convulsion was induced to all the animals (except Group I) by single dose administration of INH (250 mg/kg, i.p.) after 30 min of the treatment with the MEIF and the standard drug. All the animals were carefully monitored for 30 min for the occurrence of convulsions. The latency of convulsions was recorded and the percentage protection was calculated [25]. Thereafter, all the animals were anaesthetized and decapitated and whole brain was collected and stored at −80°C for the estimation of GABA and glutamate levels. All the animals were disposed by cremation.

*(1) Chromatographic Estimation of GABA and Glutamate in Whole Brain*. GABA and glutamate levels were estimated in rat brain using high performance liquid chromatography (HPLC, Waters, USA). Briefly, brain tissues were homogenized in 0.1 M perchloric acid containing 4 mM sodium metabisulphite solution (30 *μ*L per 10 mg of tissue) [26]. The resulting homogenate was centrifuged at 10000 rpm at 4°C for 15 min and the residual pellet was separated from the supernatant. The supernatant obtained was filtered through a syringe (Millex-HN filter, 0.45 *μ*m). Pellet and filtered supernatant were stored separately in Eppendorf tubes at −80°C until the analysis. Concentrations of GABA and glutamate were measured using precolumn derivatization with *o*-phthalaldehyde (OPA) and fluorescence was detected [27]. Derivatization was performed by mixing 20 *μ*L filtered supernatant with 6 *μ*L OPA and injecting this mixture into the solvent stream of the HPLC system, 2 min later. Separation of OPA-GABA and OPA-glutamate was carried out on a reversed phase 3.9 × 150 mm column (Nova-Pack, 4 *μ*m, C18, Waters) at 35°C using a binary gradient system of mobile phases A and B (mobile phase A: 38.74 mM sodium acetate dissolved in 90% milli-Q water and 10% methanol, pH 5.75; mobile phase B: buffer containing 20% solution A and 80% methanol, pH 6.75) at a flow rate of 0.5 mL/min. Fluorometric detection was performed with a fluorescence detector (Waters, 474) at excitation and emission wavelengths of 360 and 450 nm, respectively. This procedure allowed the quantification of GABA and glutamate levels by linear regression using external standards (Sigma, USA). Protein content was determined using the residual pellet according to a modified version of Bradford's method [28]. GABA and glutamate content in the brain tissue were expressed as *μ*g/mg protein.

#### 2.6.3. Pentylenetetrazole- (PTZ-) Induced Convulsion

In PTZ-induced convulsion model, the animals were randomly divided into six groups with six animals in each group (*n* = 6). Group I (normal control) received vehicle (0.5% CMC, p.o.) only. Group II (negative control) received PTZ (90 mg/kg, i.p.), whereas Group III (standard treated group) received vigabatrin (50 mg/kg, i.p.). Groups IV, V, and VI (MEIF treated groups) received MEIF 100, 200, and 400 mg/kg, p.o., respectively. Convulsion was induced in all the animals (except Group I) by single dose administration of PTZ (90 mg/kg, i.p.) after 30 min of the treatment with the extract or standard drug. Animals which showed no convulsions within 30 min were considered as protected and the percentage protection in each group was calculated. In unprotected animals, the latency of the first convulsion was recorded [29, 30]. All the animals were carefully monitored for 30 min for any behavioral change related to convulsion. Finally, after the observation, all the animals were anaesthetized and decapitated and the whole brain was collected for the estimation of GABA-T activity and oxidative stress markers. All the animals were disposed by cremation.

*(1) Estimation of GABA-T Activity and Oxidant Stress Markers. *500 mg of rat brain tissue was taken and rinsed with phosphate buffer solution (PBS, pH 7.2–7.4). Brain tissue was homogenized in 5.0 mL of PBS and stored overnight at −20°C. The homogenates were centrifuged for 5 min at 5000 ×*g* maintaining the temperature between 2 and 8°C. The supernatant obtained was assayed for the estimation of both GABA-T activity and oxidant stress markers.

*(1.1) Estimation of GABA-T Activity*. The supernatant of brain homogenate was assayed for GABA-T activity spectrophotometrically at 450 nm using commercially available ELISA kit (MBS939900; MyBioSource, Inc., San Diego, CA, USA). GABA-T was expressed as pg/mg protein. 

*(1.2) Estimation of LPO Activity*. Lipid peroxidation was measured and expressed in terms of malondialdehyde (MDA) as per the method of Liu et al. [31]. 1.0 mL of supernatant was added with 1.5 mL (20%, pH 3.5) acetic acid reagent, 1.5 mL thiobarbituric acid (0.8%), and 0.2 mL sodium dodecyl sulphate (8.1%). The reaction mixture was heated at 100°C for 60 min and then cooled under tap water. The mixture was then mixed with 1.0 mL of distilled water and 5.0 mL of *n*-butanol and pyridine mixture (15 : 1) and vortexed vigorously. The organic layer was separated after centrifugation at 4000 rpm for 10 min. Absorbance of organic layer was measured by using spectrophotometer at 532 nm and the concentration was expressed as nmol MDA/g tissue.

*(1.3) Estimation of GSH Activity*. Reduced glutathione activity was measured as per the method of Sedlak and Lindsay [32]. Trichloroacetic acid (50%) was added with equal quantity of brain homogenate and centrifuged at 3000 rpm for 15 min. 1.5 mL of the supernatant was mixed with 4.0 mL of 0.4 M Tris buffer and 0.1 mL of 5,5′-dithio-bis-(2-nitrobenzoic acid) (DTNB). The mixture was vigorously vortexed and the absorbance was recorded within 5 min after the addition of DTNB at 412 nm against reagent blank. The results were expressed as *μ*g GSH/g of tissue.

*(1.4) Estimation of SOD Activity*. SOD activity was determined as per the method of Kakkar et al. [33]. Brain homogenate (0.4 mL) was added with 1.2 mL, 0.052 M sodium pyrophosphate buffer (pH 8.3), 0.1 mL of 186 *μ*M phenazine methosulphate, 0.3 mL of 300 *μ*M nitroblue tetrazolium (NBT), and 0.8 mL of distilled water. This mixture was added with 2.0 mL of 780 *μ*M NADH solution and incubated at 30°C for 60 sec. After incubation, the reaction was terminated by the addition of 1.0 mL of glacial acetic acid. The reaction mixture was then shaken vigorously with 4.0 mL of *n*-butanol and later centrifuged at 3000 rpm for 5 min. The butanol layer was separated and the color intensity was measured at 560 nm against *n*-butanol. A system devoid of enzyme served as control. SOD activity in the homogenate was expressed as unit of SOD activity/g of tissue.

*(1.5) Estimation of CAT Activity*. Catalase activity was determined according to the method described by Aebi [34]. Brain homogenate (20 *μ*L) was added to a cuvette containing 2.0 mL of phosphate buffer (pH 7.0) and 1.0 mL of 30 mM H_2_O_2_. Catalase activity was measured by using spectrophotometer at 240 nm for 1 min and expressed as unit of CAT activity/g of tissue.

### 2.7. Statistical Analysis

All the results were expressed as mean ± SEM (*n* = 6). The analysis of variance was done by using one-way ANOVA followed by Tukey's posttest for multiple groups comparison. The difference was considered to be significant when *P* < 0.05. GraphPad Prism (version 5) software was used for all statistical analyses.

## 3. Results

### 3.1. Acute Toxicity Studies

MEIF at the dose of 2000 mg/kg, p.o., did not exhibit any behavioral changes or symptoms of toxicity. Hence the drug was found to be safe up to the tested dose of 2000 mg/kg, p.o.

### 3.2. Effect of MEIF on MES-Induced Convulsion

**Figure 1:**
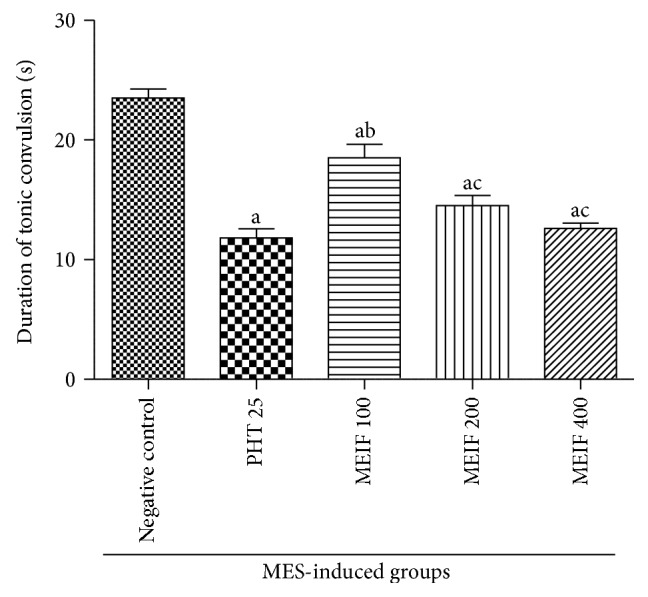
Effect of MEIF (100–400 mg/kg, p.o.) on MES-induced convulsion model in rats. Values are expressed as mean ± SEM (*n* = 6). Statistical comparison was analyzed by one-way ANOVA followed by Tukey's multiple comparison test. ^a^ *P* < 0.05, statistically significant as compared to negative control; ^b^ *P* < 0.05, statistically significant as compared to phenytoin (PHT 25 mg/kg, i.p.); ^c^ *P* < 0.05, statistically significant as compared to MEIF (100 mg/kg, p.o.).

Oral administration of MEIF (100, 200, and 400 mg/kg, p.o.) and phenytoin (25 mg/kg, i.p.) exhibited significant reduction in the duration of tonic convulsion when compared to negative control group. However, when compared to phenytoin (25 mg/kg, i.p.) treated group, MEIF (200–400 mg/kg, p.o.) exhibited no significant difference in reduction of the duration of HLTE, thus revealing that the potency of the extract is comparable to that of phenytoin (Figure 1).

### 3.3. Effect of MEIF on INH-Induced Convulsion

INH (250 mg/kg, i.p.) exhibited HLTE in all the six animals of negative control group. Diazepam (1 mg/kg, i.p.) produced 100% protection against the convulsion induced by INH. However, MEIF (200–400 mg/kg, p.o.) exhibited significant protection (up to 80%) and increased the latency of tonic convulsion as compared to negative control group (Table 1).

#### 3.3.1. Effect of MEIF on the Level of GABA and Glutamate

Rats administered with INH (250 mg/kg, i.p.) showed significant reduction in the level of GABA by 69% and elevation in the level of glutamate by 59% in the brain homogenate when compared to normal control rats. Diazepam (1 mg/kg, i.p.) and MEIF (200–400 mg/kg, p.o.) produced significant recovery in the level of GABA, whereas the level of glutamate was observed to decrease significantly. MEIF (100 mg/kg, p.o.) did not produce any significant activity in recovering the level of GABA and reduction in the level of glutamate (Table 2).

### 3.4. Effect of MEIF on PTZ-Induced Convulsion

Animal groups treated with MEIF (200–400 mg/kg, p.o.) and vigabatrin (50 mg/kg, i.p.) showed significant increase in the latency of tonic convulsions induced by PTZ (90 mg/kg, p.o.) as compared to negative control group. The percentage protection was observed to be as high as up to 66% and 100% when the rats were, respectively, treated with MEIF and standard drug vigabatrin (Table 3).

#### 3.4.1. Effect of MEIF on GABA-T Activity

**Figure 2:**
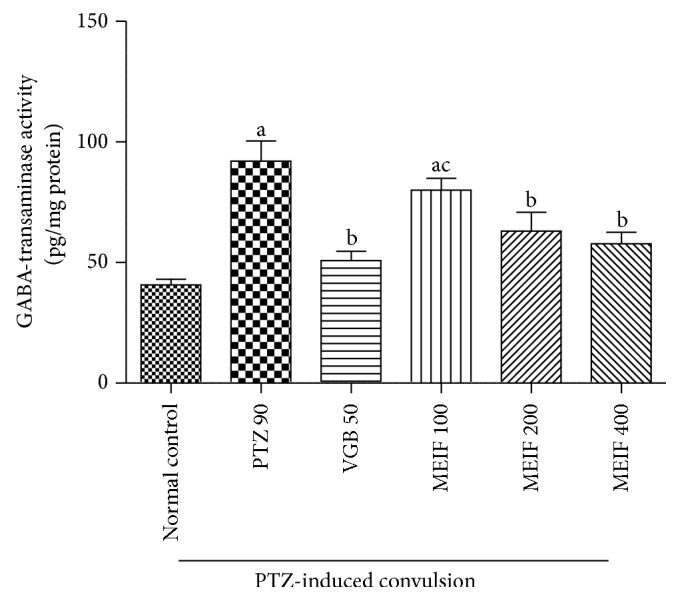
Effect of MEIF (100–400 mg/kg, p.o.) on GABA-T activity in PTZ-induced convulsion model in rats. Values are expressed as mean ± SEM (*n* = 6). Statistical comparison was analyzed by one-way ANOVA followed by Tukey's multiple comparison test. ^a^ *P* < 0.05, statistically significant as compared to normal control; ^b^ *P* < 0.05, statistically significant as compared to negative control (PTZ 90 mg/kg, i.p.); ^c^ *P* < 0.05, statistically significant as compared to vigabatrin (VGB 50 mg/kg, i.p.).

Rats administered with PTZ (90 mg/kg, i.p.) showed significant increase in the level of GABA-T enzymatic activity by 46% as compared to normal control group. Treatment of the animals with standard drug vigabatrin (50 mg/kg, i.p.) and MEIF (200–400 mg/kg, p.o.) produced a significant reduction in the level of GABA-T enzyme when compared to negative control group. However, the lower dose of MEIF (100 mg/kg, p.o.) showed no significant effect in the reduction of enzyme activity (Figure 2).

#### 3.4.2. Effect of MEIF on the Level of Oxidative Stress Markers

The results in Table 4 depict that rats administered with PTZ (90 mg/kg, i.p.) showed significant increase in the level of LPO, while significant reduction in the level of SOD, GSH, and CAT was observed as compared to normal control group. Diazepam (1 mg/kg, i.p.) and MEIF (400 mg/kg, p.o.) significantly produced an excellent reduction in the level of LPO and increased the level of SOD, GSH, and CAT significantly. MEIF (200 mg/kg, p.o.) also showed a similar effect but was found to be ineffective in restoring the levels of SOD. However, MEIF (100 mg/kg, p.o.) showed a significant recovery in the levels of LPO and GSH, but no effect was observed on SOD and CAT levels (Table 4).

## 4. Discussion

The objective of the present study was to investigate the protective effect of MEIF (100, 200, and 400 mg/kg, p.o.) against physical and chemical induced convulsion in experimental rat models. MEIF (200 and 400 mg/kg, p.o.) dose-dependently reduced the convulsions in MES treated rats. Pretreatment of MEIF (200 and 400 mg/kg, p.o.) reversed INH-induced decrease and increase in the level of GABA and glutamate in the rat brain, respectively. MEIF (200 and 400 mg/kg, p.o.) attenuated the PTZ-induced increase in the activity of GABA-T in the rat brain. Further, MEIF (200 and 400 mg/kg, p.o.) reversed the PTZ-induced increase in the level of LPO and decrease in GSH, CAT, and SOD levels.

Root of* I. frutescens* is an important ingredient of many Ayurvedic formulations, commonly used for the treatment of epilepsy along with other diseases, but has not been investigated scientifically. In MES-induced convulsion model, neither phenytoin nor MEIF (200 and 400 mg/kg, p.o.) protected the animal completely, but a significant reduction in the duration of tonic convulsion was observed. Lower dose of MEIF (100 mg/kg, p.o.) showed no significant reduction in the duration of convulsion in MES exposed animals. MES induces seizure particularly due to the spread of stimulus throughout the body and anticonvulsant drugs that block the effect of MES act by blocking the seizure spread [35]. Thus, the present study indicates that MEIF (200 and 400 mg/kg, p.o.) has significant ability to slow down the spread of seizure.

Epilepsy is a condition of spontaneous recurrent seizures having a close relation with the GABA_A_ receptor [36]. Seizure is the result of the imbalance between excitatory and inhibitory neurotransmitters in the brain [37]. GABA and glutamate are, respectively, the most important inhibitory and excitatory neurotransmitters located in the mammalian brain [5]. Glutamic acid decarboxylase (GAD) is a key enzyme in the biogenesis of GABA from glutamic acid in the neurons, thereby maintaining a balance between GABA and glutamate. It is well known that INH-induced seizure is attributed mainly to the inhibition of the enzyme GAD in the neurons that ultimately lead to the depletion and elevation in the level of GABA and glutamate in the brain, respectively [38]. Drugs that decrease the activity of glutamatergic system and/or increase the activity of GABAergic system are beneficial in the therapeutic strategy of epilepsy. Therefore, the levels of GABA and glutamate were estimated in INH-induced convulsion model of epilepsy to elucidate the effect of MEIF (100, 200, and 400 mg/kg, p.o.) on these two neurotransmitters' activity. In the present study, INH (250 mg/kg, i.p.) significantly decreased and increased the level of GABA and glutamate, respectively, in the rat brain. Diazepam (1 mg/kg, i.p.) and MEIF (200 and 400 mg/kg, p.o.) significantly reversed the INH-induced changes in the levels of both GABA and glutamate in rat brain. This indicates that MEIF (200 and 400 mg/kg, p.o.) may probably be able to retain the balance between GABA and glutamate levels during seizure.

As MEIF (200 and 400 mg/kg, p.o.) reversed the INH-induced decrease in the GABAergic activity, we further investigated the effect of MEIF (100, 200, and 400 mg/kg, p.o.) in PTZ-induced animal model of convulsion. It is well reported that PTZ induces convulsion through antagonism at the picrotoxin-sensitive site of GABA_A_ receptor complex [39]. As a result, the glutamatergic activity predominates over GABAergic activity which leads to convulsion in the animals. In addition, it has also been reported that PTZ increases the level of GABA-T in the brain [40]. Drugs showing protective effect against the PTZ-induced tonic clonic convulsion are considered in the treatment of myoclonic and absence seizure in humans [41].

In the present study, standard drug vigabatrin (50 mg/kg, i.p.) showed complete protection against PTZ-induced convulsion while MEIF (200 and 400 mg/kg, p.o.) showed dose-dependent effect against PTZ-induced convulsion. GABA-T is the primary catabolic enzyme in the mammalian brain that catalyzes the transfer of amino group from GABA to *α*-ketoglutarate leading to the depletion in the level of GABA [42]. PTZ significantly increased the level of GABA-T activity in the rat brain. Administration of MEIF (200–400 mg/kg, p.o.) and vigabatrin (50 mg/kg, i.p.) significantly attenuated the enzyme activity indicating potent GABA-T inhibitory effect. It is well studied that free radicals are generated during convulsion and disturb the balance between GABA and glutamate activity in the brain [43, 44]. Thus, in addition to GABA-T inhibitory activity, we have attempted to estimate the markers of oxidative damage in the brain of PTZ exposed rats. PTZ (90 mg/kg, i.p.) significantly increased the activity of LPO enzyme and reduced the activity of antioxidant enzymes such as SOD, CAT, and GSH in the rat brain. Both standard drug and MEIF (200 and 400 mg/kg, p.o.) decreased the PTZ-induced increase in the activity of LPO. On the contrary, both increased the PTZ-induced decrease in the activities of SOD, GSH, and CAT in the brain. This indicates that MEIF (200 and 400 mg/kg, p.o.) has profound antioxidant activity in convulsive rats.

Literature survey reveals that MEIF is a rich source of triterpenoids, phenolics, and steroids [12]. Recently, *α*-amyrin and ursolic acid have been isolated from MEIF and standardized by HPTLC [45].* I. frutescens *is also reported to contain quercetin along with other phenolics [12]. It has been reported that ursolic acid (triterpenoid) has significant GABA-T inhibitory activity in* in vitro* study [46] and protects against the PTZ-induced convulsion in rats [47]. Quercetin, a phenolic component, has been reported to reduce the seizure and duration of convulsion in amygdala electrical kindling model in rats [48]. Moreover, *α*-amyrin (triterpenoid) retains a balance between excitatory and inhibitory neurotransmitters in convulsion [49]. Phenolics are also well known for their antioxidant activity in convulsive animals. Thus, the polyphenols in the MEIF may be responsible for the anticonvulsant activity through modulating GABAergic system and oxidative stress in the brain.

## 5. Conclusion

Hence, from the present study, it can be concluded that MEIF (200–400 mg/kg, p.o.) showed anticonvulsant activity through modulating GABAergic system and oxidative stress in the rat's brain which can be predicted due to the presence of different category of phytoconstituents (phenolics, terpenoids, and steroids). Therefore, roots of* I. frutescens *could be a potential source in the management of convulsion.

## Figures and Tables

**Table 1:** Effect of MEIF graded dose on INH-induced convulsion in rats.

Treatments dose			Number convulsed/number used	% Animals protected	Latency of tonic convulsion (min) (mean ± SEM)
INH (mg/kg, i.p.)	Diazepam (mg/kg, i.p.)	MEIF (mg/kg, p.o.)			
250	—	—	6/6	0	13.20 ± 1.00
250	—	100	5/6	16.67	17.04 ± 0.59
250	—	200	3/6	50	20.55 ± 0.80^a^
250	—	400	1/6	83.33	27.75 ± 2.55^abc^
250	1.0	—	0/6	100	∞^a^

Values are expressed as mean ± SEM (*n* = 6). Statistical comparison was analyzed by one-way ANOVA followed by Tukey's multiple comparison test. ^a^ *P* < 0.05, statistically significant as compared to negative control; ^b^ *P* < 0.05, statistically significant as compared to MIEF (100 mg/kg, p.o.); ^c^ *P* < 0.05, statistically significant as compared to MEIF (200 mg/kg, p.o.).

**Table 2:** Effect of MEIF graded dose on GABA and glutamate level in brain homogenate.

Treatments dose			GABA (*µ*g/mg protein)	Glutamate (*μ*g/mg protein)
INH (mg/kg, i.p.)	Diazepam (mg/kg, i.p.)	MEIF (mg/kg, p.o.)		
—	—	—	33.13 ± 1.22	52.38 ± 1.57
250	—	—	10.21 ± 0.92^a^	126.27 ± 15.25^a^
250	1.0	—	25.17 ± 3.96^b^	66.63 ± 1.20^b^
250	—	100	15.63 ± 3.50^a^	105.15 ± 10.49^ac^
250	—	200	24.01 ± 3.71^b^	82.58 ± 4.65^b^
250	—	400	25.60 ± 1.16^b^	74.67 ± 3.23^b^

Values are expressed as mean ± SEM (*n* = 6). Statistical comparison was analyzed by one-way ANOVA followed by Tukey's multiple comparison test. ^a^ *P* < 0.05, statistically significant as compared to normal control; ^b^ *P* < 0.05, statistically significant as compared to negative control (INH); ^c^ *P* < 0.05, statistically significant as compared to diazepam 1.0 mg/kg, i.p.

**Table 3:** Effect of MEIF graded dose on pentylenetetrazole- (PTZ-) induced convulsion in rats.

Treatments dose			Number convulsed/number used	% Animals protected	Latency of tonic convulsion (min) (mean ± SEM)
PTZ (mg/kg, i.p.)	Vigabatrin (mg/kg, i.p.)	MEIF (mg/kg, p.o.)			
90	—	—	6/6	0	10.7 ± 0.34
90	—	100	5/6	16.67	12.2 ± 0.41
90	—	200	4/6	33.33	13.0 ± 0.55^a^
90	—	400	0/6	66.67	16.6 ± 0.65^abc^
90	50	—	0/6	100	∞^a^

Values are expressed as mean ± SEM (*n* = 6). Statistical comparison was analyzed by one-way ANOVA followed by Tukey's multiple comparison test. ^a^ *P* < 0.05, statistically significant as compared to negative control; ^b^ *P* < 0.05, statistically significant as compared to MIEF (100 mg/kg, p.o.); ^c^ *P* < 0.05, statistically significant as compared to MEIF (200 mg/kg, p.o.).

**Table 4:** Effect of MEIF graded dose on pentylenetetrazole- (PTZ-) induced convulsion in rats.

Treatments dose			LPO (nmole MDA/g brain tissue)	SOD (unit/g brain tissue)	CAT (unit/g brain tissue)	GSH (*μ*g/g brain tissue)
PTZ (mg/kg, i.p.)	Vigabatrin (mg/kg, i.p.)	MEIF (mg/kg, p.o.)				
—	—	—	20.12 ± 0.82	30.53 ± 0.48	25.02 ± 0.65	256.4 ± 19.24
90	—	—	52.87 ± 0.96^a^	15.13 ± 3.03^a^	11.47 ± 1.12^a^	121.02 ± 14.20^a^
90	50	—	23.17 ± 1.83^b^	26.70 ± 1.92^b^	23.14 ± 1.30^b^	242.0 ± 11.98^b^
90	—	100	43.29 ± 2.72^abc^	18.27 ± 1.71^a^	15.35 ± 0.83^ac^	192.57 ± 12.75^b^
90	—	200	38.62 ± 2.81^abc^	21.56 ± 2.35^a^	19.85 ± 0.76^abd^	218.90 ± 17.80^b^
90	—	400	28.28 ± 2.2^bde^	25.75 ± 1.60^b^	21.73 ± 1.02^bd^	235.86 ± 13.31^b^

Values are expressed as mean ± SEM (*n* = 6). Statistical comparison was analyzed by one-way ANOVA followed by Tukey's multiple comparison test. ^a^ *P* < 0.05, statistically significant as compared to normal control; ^b^ *P* < 0.05, statistically significant as compared to negative control (PTZ 90); ^c^ *P* < 0.05, statistically significant as compared to standard vigabatrin (50 mg/kg, i.p.); ^d^ *P* < 0.05, statistically significant as compared to MEIF (100 mg/kg, p.o.); ^e^ *P* < 0.05, statistically significant as compared to MEIF (200 mg/kg, p.o.).
